# Phosphate homeostasis and apparent treatment-resistant hypertension in chronic kidney disease: the Chronic Renal Insufficiency Cohort (CRIC) study

**DOI:** 10.1038/s41440-026-02706-5

**Published:** 2026-06-17

**Authors:** Alvin G. Kwon, Jiang He, L. Lee Hamm, Arnold Alper, Siyi Geng, Paola Lanza, Haowen Qin, Hua He, Halil K. Erol, Suayp Oygen, Joshua D. Bundy, Eleanor Lederer, Robert Toto, Jing Chen

**Affiliations:** 1 Department of Internal Medicine, Division of Nephrology, University of Texas Southwestern Medical Center, Dallas, TX USA; 2 Charles and Jane Pak Center of Mineral Metabolism and Clinical Research, University of Texas Southwestern Medical Center, Dallas, TX USA; 3 Department of Epidemiology, O’Donnell School of Public Health, University of Texas Southwestern Medical Center, Dallas, TX USA; 4 Department of Neurology, University of Texas Southwestern Medical Center, Dallas, TX USA; 5 Department of Medicine, Tulane University School of Medicine, New Orleans, LA USA; 6 Department of Epidemiology, Tulane University School of Public Health and Tropical Medicine, New Orleans, LA USA; 7 Division of Nephrology, Department of Medicine, University of Arizona College of Medicine, Tucson, AZ USA; 8 Nephrology Department, University of Utah School of Medicine, Salt Lake City, UT USA; 9 Medical Service, VA North Texas Health Care Services, Dallas, TX USA; 10 University of Alabama at Birmingham, Birmingham, AL USA; 11 Johns Hopkins University School of Medicine, Baltimore, MD USA; 12 University of Texas Southwestern Medical Center, Dallas, TX USA; 13 Perelman School of Medicine, University of Pennsylvania, Philadelphia, PA USA; 14 Kaiser Permanente Northern California, Oakland, CA USA; 15 Tulane University School of Medicine, New Orleans, LA USA; 16 University of Illinois Chicago College of Medicine, Chicago, IL USA; 17 Case Western Reserve University School of Medicine, Cleveland, OH USA; 18 University of Michigan Medical School, Ann Arbor, MI USA

**Keywords:** Apparent treatment-resistant hypertension, Chronic kidney disease, Parathyroid hormone, Phosphate homeostasis, Serum phosphate

## Abstract

Abnormal phosphate homeostasis is associated with inflammation, vascular calcification, and endothelial dysfunction in chronic kidney disease (CKD). Apparent treatment-resistant hypertension (ATRH) is common in CKD and is associated with increased cardiovascular mortality. We examined the associations between phosphate homeostasis-related biomarkers and ATRH in patients with CKD. The Chronic Renal Insufficiency Cohort (CRIC) Study enrolled 3939 participants with CKD in the United States between 2003 and 2008. After excluding those with missing ATRH data, 3752 were analyzed. ATRH was defined as blood pressure (BP) ≥ 140/90 mm Hg while taking ≥3 antihypertensive medications, or BP < 140/90 mm Hg while taking ≥4 medications. In addition to phosphate homeostasis-related biomarkers, we calculated the phosphate burden index as the combined effect of phosphate retention on circulation and bone metabolism: index = *serum phosphate × parathyroid hormone*. Elastic Net and multivariable regression models assessed associations between phosphate biomarkers and ATRH. Participants with ATRH were older and more often male and Black. The adjusted odds ratios (95% confidence intervals) for the highest quartiles compared with the lowest quartile were 1.42 (1.09–1.86) for serum phosphate, 1.48 (1.15–1.91) for the urine phosphate-to-creatinine ratio, and 2.30 (1.72–3.08) for phosphate burden index. These associations were also significant in linear regression models for the urine phosphate-to-creatinine ratio and the phosphate burden index. We conclude that higher levels of phosphate-related biomarkers are independently associated with ATRH. Future studies should investigate whether targeting abnormal phosphate homeostasis can reduce ATRH risk in patients with CKD.

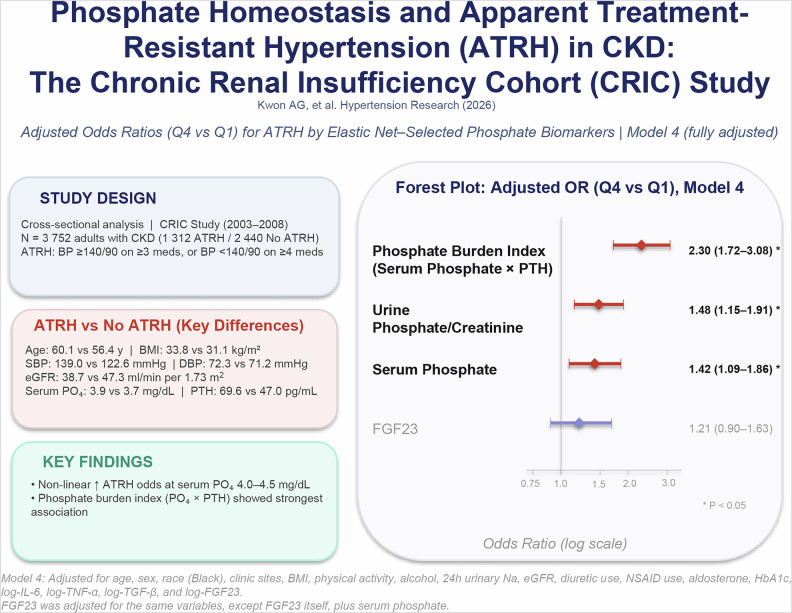

## Introduction

The prevalence of apparent treatment-resistant hypertension (ATRH) is approximately 28–40% in individuals with chronic kidney disease (CKD), compared with about 12–15% in the general US population [1–4]. This phenotype is significantly associated with increased risks of cardiovascular disease (CVD), mortality, and CKD progression [2, 5–7]. However, the mechanisms underlying the disproportionately high prevalence of ATRH in CKD remain incompletely understood.

Abnormal phosphate homeostasis is prevalent [8, 9], and hyperphosphatemia is an established risk factor for adverse cardiovascular outcomes across the CKD spectrum, from non-end-stage kidney disease (non-ESKD) to ESKD patients [10, 11]. Preclinical studies show that high phosphate intake increases fibroblast growth factor 23 (FGF23) and activates the skeletal muscle exercise pressor reflex and the renin-angiotensin system (RAS), contributing to elevated blood pressure (BP) [12–14]. Clinical studies report associations between elevated serum phosphate or FGF23 levels and hypertension, as well as endothelial dysfunction, in both CKD and non-CKD populations [15–20]. Higher phosphate levels are associated with vascular calcification and atherosclerotic diseases, key contributors to arterial stiffness and cardiovascular risk [21]. Although phosphate retention and its downstream effects (“phosphate burden”) have been implicated in cardiovascular complications [10, 11, 22–25], the association between abnormal phosphate homeostasis and ATRH has not been well studied in patients with CKD. No prior study has examined phosphate homeostasis in relation to ATRH in patients with CKD. This study aimed to examine the association between phosphate homeostasis biomarkers and ATRH in patients with CKD in the Chronic Renal Insufficiency Cohort (CRIC) Study [26].

## Methods

### Study design

This cross-sectional analysis evaluated associations between abnormal phosphate homeostasis biomarkers and ATRH among adults with CKD enrolled in the Chronic Renal Insufficiency Cohort (CRIC) Study [27].

### Study participants

The CRIC Study enrolled a racially and ethnically diverse cohort of men and women aged 21–74 years with mild-to-moderate CKD (age-based estimated-glomerular filtration rate [eGFR] entry criteria 20–70 ml/min/1.73 m^2^) [26]. In total, 3939 participants were recruited between May 2003 and August 2008 from seven US clinical centers. Participants were identified through laboratory databases, medical records, and clinician referrals. Patients with cirrhosis, HIV infection, polycystic kidney disease, or renal cell carcinoma; those on dialysis or recipients of a kidney transplant; or those taking immunosuppressant drugs for glomerulonephritis were excluded [26, 28]. After excluding individuals with missing ATRH data at baseline, 3 752 participants were included in the analytic sample. The CRIC Study was approved by the Institutional Review Boards from each participating institute and was conducted in accordance with the Declaration of Helsinki. Written informed consent was obtained from all participants. The study complied with Health Insurance Portability and Accountability Act (HIPAA) requirements.

### Data collection

Baseline and annual clinical data were collected by trained study staff using standardized procedures. Baseline demographic, lifestyle, medical history, and medication data were obtained using standardized questionnaires. Cigarette smokers were defined as participants who smoked ≥100 cigarettes in their lifetime and reported currently smoking, and alcohol drinkers as those who consumed one or more alcoholic beverages each week over the previous year. Physical activity was calculated as the total metabolic equivalents (METs) per week. Body weight and height were measured, and body mass index (BMI) was calculated as weight in kilograms divided by height in meters squared. Three seated BP measurements were obtained after at least 5 min of rest using an aneroid sphygmomanometer, and the average of the three readings was used for analysis [1]. Hypertension was defined as systolic BP (SBP) ≥140 mm Hg, diastolic BP (DBP) ≥90 mm Hg, or current use of antihypertensive medication. Diabetes was defined as a fasting plasma glucose ≥126 mg/dL, a non-fasting plasma glucose ≥200 mg/dL, or self-reported use of anti-diabetes medication.

Glucose, cholesterol, triglycerides, glycated hemoglobin (HbA1c), serum phosphate, urine phosphate, and urine sodium were measured using standard laboratory methods. FGF23 was measured using a second-generation C-terminal assay (Immutopics, San Clemente, CA; coefficient of variation (CV) < 5%). Parathyroid hormone (PTH) was measured using a total PTH assay, which includes the 1-84 PTH molecule and 7-84 fragments (Scantibodies, Santee, CA; CV < 5%). High-sensitivity sandwich ELISAs (Quantikine HS; R&D Systems, Minneapolis, MN) were used to measure plasma interleukin-6 (IL-6) and tumor necrosis factor alpha (TNF-α) levels. Standard sandwich ELISAs (Quantikine; R&D Systems) were used to quantify transforming growth factor beta (TGF-β) levels. eGFR was calculated using the CKD-EPI (Chronic Kidney Disease Epidemiology Collaboration) equation after calibration of serum creatinine to isotope dilution mass spectrometry standards [29]. All laboratory measurements were conducted at the CRIC Study central laboratory at the University of Pennsylvania with stringent quality control.

### Definition of ATRH

ATRH was defined as mean SBP ≥ 140 mm Hg or mean DBP ≥ 90 mm Hg while taking ≥3 antihypertensive medications, or mean SBP < 140 mm Hg and mean DBP < 90 mm Hg while taking ≥4 antihypertensive medications at the baseline visit [2, 5]. A sensitivity analysis applied the 2025 American College of Cardiology/American Heart Association (ACC/AHA) hypertension guideline: mean SBP ≥ 130 mm Hg or mean DBP ≥ 80 mm Hg while taking ≥3 antihypertensive medications, including a diuretic, mean SBP < 130 mm Hg and mean DBP < 80 mm Hg while taking ≥4 antihypertensive medications [30].

### Phosphate burden definition, rationale and phosphate indices

“Phosphate burden” was defined as the degree of disturbance in phosphate homeostasis—encompassing PTH, FGF23, serum phosphate levels, and renal phosphate handling—reflect the cumulative physiological stress from phosphate retention and compensatory hormonal responses, rather than serum phosphate alone [31, 32]. To capture phosphate homeostasis across serum, kidney, and bone compartments, phosphate burden indices were constructed using multiplicative interactions among serum phosphate, alkaline phosphatase (ALP), PTH, FGF23, 24-h urinary phosphate, urine phosphate-to-creatinine ratio, and fractional excretion of phosphate (FEPO_4_), which are typically elevated in the setting of phosphate excess or maladaptive homeostatic responses [33, 34].

$$ {{{{\rm{FEPO}}}}}_{4}= \quad \frac{{{{\rm{urine}}}}\; {{{\rm{phosphate}}}}\; {{{\rm{from}}}}\,24{{{\rm{hour}}}}\; {{{\rm{sample}}}}/{{{\rm{serum}}}}\; {{{\rm{phosphate}}}}}{{{{\rm{urine}}}}\; {{{\rm{creatinine}}}}\; {{{\rm{from}}}}\,24{{{\rm{hour}}}}{{{\rm{sample}}}}/{{{\rm{serum}}}}\; {{{\rm{creatinine}}}}}$$

Based on physiological rationale, we examined three candidate indices representing the combined effects of circulating phosphate, bone or endocrine responses, and renal phosphate excretion: *serum phosphate × PTH × FEPO*_*4*_, *serum phosphate × ALP × FEPO*_*4*_, and *serum phosphate × PTH*. Because serum phosphate is tightly regulated and often remains within the normal range in early CKD, these composite indices were designed to reflect systemic phosphate dysregulation that may not be apparent from individual biomarkers alone. All indices were considered exploratory constructs and have not undergone external validation.

### Statistical methods

#### Baseline characteristics

Baseline characteristics of participants were summarized as means (standard deviation [SD]) for continuous variables, percentages for categorical variables, or medians (interquartile range [IQR]) for variables with skewed distribution, by ATRH status. Statistical significance was tested using a two-sample t-test for continuous variables and χ2 tests for categorical variables. Logarithmic (log) transformations were performed for severely skewed variables to stabilize variances and normalize distributions. Because several biomarkers and indices were skewed, log‑transformation was applied before modeling; per‑SD effects for log‑transformed variables refer to the SD on the log scale. Also, because subgroup and interaction analyses were exploratory, P values for interaction were not adjusted for multiple comparisons and should be interpreted as hypothesis‑generating.

#### Variable selection

Penalized regression was used to evaluate phosphate-related biomarkers while addressing multicollinearity [35]. Candidate predictors included serum phosphate, PTH, FGF23, FEPO_4_, 24-h urine phosphate, 24-h urine phosphate-to-creatinine ratio, ALP, and phosphate burden indices ([*serum phosphate × PTH × FEPO*_*4*_], [*serum phosphate × ALP × FEPO*_*4*_], and [*serum phosphate × PTH*]). Based on prior literature establishing their associations with hypertension, we included age, sex, race (Black), clinical sites, BMI, eGFR, and diuretic use as unpenalized (forced) covariates. We also forced physical activity, weekly alcohol intake, 24‑h urinary sodium, NSAID use, serum aldosterone, HbA1c, and inflammatory biomarkers (IL‑6, TNF‑α, TGF‑β) into the model to ensure consistent adjustment across analyses. Elastic Net logistic regression with ten-fold cross-validation was used to select predictors with non-zero coefficients at λmin, incorporating both candidate and unpenalized covariables [35].

#### Association analyses

Logistic regression models were used to explore the cross-sectional association of the levels of serum phosphate, urine phosphate-to-creatinine ratio, FGF23, phosphate burden index and odds of ATRH by quartiles or one SD increment of the biomarkers. The logistic regression models include Model 1: age, sex, race/ethnicity, and clinical sites; Model 2: Model 1 plus BMI, physical activity, weekly drinking, 24-h urinary sodium, eGFR, use of diuretic, use of non-steroidal anti-inflammatory drugs (NSAIDs), serum aldosterone, and HbA1c; Model 3: Model 2 plus log-IL-6, log-TNF-α, and log-TGF-β; and Model 4: Model 3 plus log-FGF23 for the exposure biomarkers other than FGF23 or Model 3 plus serum phosphate for exposure FGF23. Subgroup analyses stratified participants by eGFR cut off 30 ml/min per 1.73 m^2^ to identify phosphate biomarkers associated with ATRH in earlier CKD stages, where serum phosphate levels typically remain within normal range [36]. Non-linear associations were modeled using restricted cubic splines for continuous predictors. A sensitivity analysis applied the 2025 ACC/AHA definition of resistant hypertension [30]. All analyses were conducted using SAS version 9.4 (SAS Institute, Inc) and R version 3.6.0 (The R Foundation). All P values were 2-sided, and statistical significance was defined as *P* < 0.05. Artificial Intelligence-assisted tools (Microsoft Copilot [Microsoft Corporation, Redmond, WA, USA] and Claude [Anthropic, San Francisco, CA, USA]) were used for language editing.

## Results

Baseline characteristics of the 1312 CRIC Study participants with ATRH and 2440 without ATRH are shown in Table 1. Participants with ATRH were older, more often male and Black, and had a lower level of education, physical activity, and alcohol intake, along with a higher prevalence of CVD (myocardial infarction, stroke, congestive heart failure, and peripheral artery disease), and diabetes mellitus. Use of RAS blockers, β-blockers, statins, and aspirin was more common among participants with ATRH compared to those without. Participants with ATRH also had higher BMI, waist circumference, systolic and diastolic BP, serum aldosterone, ALP, total PTH, FGF23, urine albumin to creatinine ratio from 24-h urine, FEPO_4_, serum IL-6 and TNF-α levels, and a higher prevalence of eGFR <30 ml/min per 1.73 m^2^, along with lower eGFR, HDL-cholesterol, LDL-cholesterol, and TGF-β levels.

**Table 1:** Baseline Characteristics of the Study Participants by Apparent Treatment-Resistant Hypertension Status

Variables	No Apparent Treatment-Resistant Hypertension (*n* = 2 440)	Apparent Treatment-Resistant Hypertension (*n* = 1312)	*P* value
Age, mean (SD), years	56.4 (11.6)	60.1 (9.2)	<.001
Male, no. (%)	1272 (52.1)	767 (58.5)	<.001
Race/ethnicity, Black (%)	841 (34.5)	711 (54.2)	<.001
High school education (%)	2006 (82.2)	974 (74.2)	<.001
Physical activity, mean (SD), total metabolic equivalent	210.3 (150.8)	177.7 (129.5)	<.001
Current smoking (%)	327 (13.4)	155 (11.8)	0.182
Weekly drinking more than once (%)	1658 (68.0)	699 (53.3)	<.001
Diabetes mellitus (%)	939 (38.5)	852 (64.9)	<.001
History of cardiovascular disease^a^ (%)	561 (23.0)	673 (51.3)	<.001
Use of statin medication (%)	1184 (48.5)	882 (67.2)	<.001
Use of other lipid-lowering medications (%)	302 (12.4)	206 (15.7)	0.005
Use of aspirin (%)	907 (37.2)	692 (52.7)	<.001
Use of antihypertensive medications (%)	2130 (87.3)	1312 (100.0)	<.001
Angiotensin converting enzyme inhibitors or angiotensin receptor blockers	1453 (59.5)	1120 (85.4)	<.001
Beta blockers	764 (31.3)	1074 (81.9)	<.001
Body-mass index, mean (SD), kg/m^2^	31.1 (7.6)	33.8 (7.8)	<.001
Waist circumference, mean (SD), cm	103.4 (17.2)	110.3 (17.2)	<.001
Systolic blood pressure, mean (SD), mm Hg	122.6 (18.6)	139.0 (24.2)	<.001
Diastolic blood pressure, mean (SD), mm Hg	71.2 (11.7)	72.3 (14.6)	0.023
eGFR (2021 CKD-EPI), mean (SD), ml/min per 1.73 m^2^	47.3 (15.7)	38.7 (12.6)	<.001
eGFR ≥30 ml/min per 1.73 m^2^ (%)	2093 (85.8)	951 (72.5)	<.001
Total cholesterol, mean (SD), mg/dL	186.3 (45.2)	179.5 (45.9)	<.001
High-density lipoprotein cholesterol, mean (SD), mg/dL	48.9 (16.1)	45.5 (14.2)	<.001
Low-density lipoprotein cholesterol, mean (SD), mg/dL	105.0 (35.1)	99.1 (36.0)	<.001
Serum aldosterone, median [IQR], (pg/mL)	98.9 [69.1,150.7]	109.2 [76.2,157.5]	<.001
Serum phosphate, mean (SD), mg/dL	3.7 (0.6)	3.9 (0.7)	<.001
Serum creatinine, mean (SD), mg/dL	1.7 (0.6)	2.0 (0.7)	<.001
Alkaline phosphatase, median [IQR], U/L	82.0 [69.0, 102.0]	89.0 [73.0, 108.0]	<.001
PTH, median [IQR], pg/mL	47.0 [32.0, 76.0]	69.6 [44.3, 115.0]	<.001
Fibroblast growth factor-23, median [IQR], RU/mL	128.9 [87.6, 205.9]	176.5 [115.6, 294.7]	<.001
Urine phosphate concentration from 24-h urine, mean (SD), mg/dL	40.5 (20.4)	36.8 (16.1)	<.001
Urine phosphate excretion, mean (SD), mg/24-h	762.3 (353.0)	741.2 (339.5)	0.083
Urine creatinine concentration from 24-h urine, mean (SD), mg/dL	72.8 (38.7)	67.2 (33.9)	<.001
Urine phosphate to urine creatinine ratio, median [IQR], mg/mg	0.57 [0.44, 0.73]	0.56 [0.43, 0.73]	0.810
Urine albumin to creatinine ratio from 24-h urine, median [IQR], mg/g	27.1 [6.4, 263.9]	170.8 [23.0, 925.1]	<.001
Fractional excretion of phosphate, median [IQR]	0.25 [0.19, 0.35]	0.29 [0.21, 0.39]	<.001
eGFR <30 ml/min per 1.73 m^2^	0.37 [0.28, 0.50]	0.36 [0.27, 0.48]	0.499
eGFR ≥30 ml/min per 1.73 m^2^	0.24 [0.18, 0.33]	0.26 [0.20, 0.36]	<.001
Phosphate burden index (serum phosphate × PTH), median [IQR]	166.6 [112.0, 277.2]	261.8 [163.4, 448.5]	<.001
Interleukin-6, median [IQR], pg/mL	1.7 [1.0, 2.9]	2.2 [1.5, 3.5]	<.001
Tumor necrosis factor-α, median [IQR], pg/mL	2.1 [1.4, 3.1]	2.5 [1.7, 3.6]	<.001
Transforming growth factor-β, median [IQR], ng/mL	11.2 [6.5, 18.2]	10.3 [6.3, 17.1]	0.031

*SD* standard deviation, *eGFR* estimated glomerular filtration rate, *IQR* interquartile range, *PTH* parathyroid hormone

Apparent Treatment-Resistant Hypertension was defined as mean SBP ≥ 140 mm Hg or mean DBP ≥ 90 mm Hg while taking ≥3 antihypertensive medications or mean SBP < 140 mm Hg and mean DBP < 90 mm Hg while taking ≥4 antihypertensive medications at the baseline visit

^a^Cardiovascular disease was defined as myocardial infarction, stroke, congestive heart failure, and peripheral artery disease

The Elastic Net models included two composite indices incorporating renal phosphate handling (*serum phosphate × PTH × FEPO*_*4*_ and *serum phosphate × ALP × FEPO*_*4*_), as well as a simpler phosphate burden index (*serum phosphate × PTH*) that captures the combined effects of phosphate retention on the circulation and bone metabolism, along with other covariates. These models consistently selected serum phosphate, FGF23, the urine phosphate-to-creatinine ratio, and the phosphate burden index (*serum phosphate × PTH*) as predictors.

**Fig. 1:**
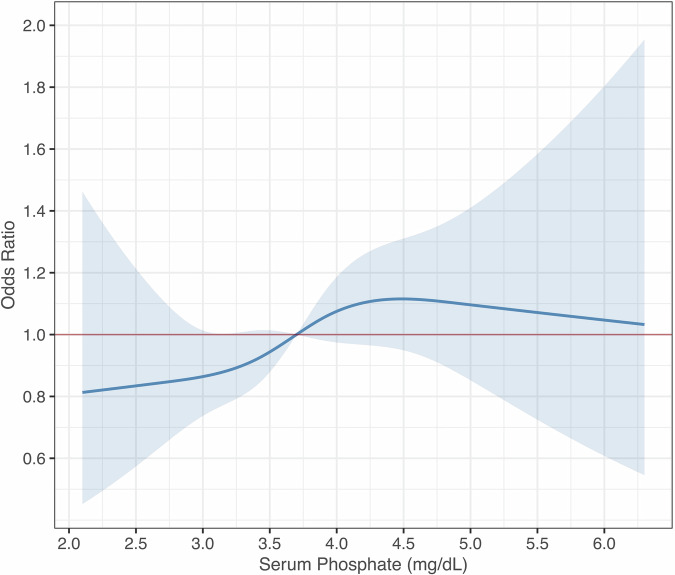
Odds Ratios (95% Confidence Intervals) for Apparent Treatment-Resistant Hypertension Associated with Serum Phosphate Levels. Adjusted for age, sex, Black, clinic sites, BMI, physical activity, weekly drinking, 24-h urinary sodium, eGFR (2021 CKD-EPI), use of diuretic, use of NSAID, serum aldosterone, Hemoglobin A1c, log-IL-6, log-TNF-alpha, log-TGF-beta, and log-FGF23. Apparent Treatment-Resistant Hypertension was defined as mean SBP ≥ 140 mm Hg or mean DBP ≥ 90 mm Hg while taking ≥3 antihypertensive medications or mean SBP < 140 mm Hg and mean DBP < 90 mm Hg while taking ≥4 antihypertensive medications at the baseline visit

Table 2 presents multivariable-adjusted odds ratios (ORs) (95% confidence intervals [CI]) for ATRH by quartiles and per-SD increments of phosphate-related biomarkers and phosphate burden index. In Model 4, the highest quartile versus the lowest quartile of serum phosphate was associated with higher odds of ATRH (OR 1.42; 95% CI, 1.09–1.86). Figure 1 illustrates a non-linear association between serum phosphate and the odds of ATRH. It illustrates the restricted cubic spline for serum phosphate, showing a relatively flat association at lower levels and a marked increase in ATRH odds beginning around 4.0–4.5 mg/dL.

**Table 2:** Multivariable-adjusted Odds Ratios for Apparent Treatment-Resistant Hypertension by Quartiles and Per Standard Deviation Increments of Phosphate-Related Biomarkers and Phosphate Burden Index

	Numbers of Participants with Apparent Treatment-resistant Hypertension (%)	Model 1		Model 2		Model 3		Model 4	
		OR (95% CI)	*P* value	OR (95% CI)	*P* value	OR (95% CI)	P value	OR (95% CI)	P value
Serum phosphate, mg/dL									
Quartile 1, <3.3	249 (27.9)	1 (Reference)		1 (Reference)		1 (Reference)		1 (Reference)	
Quartile 2, 3.3 to <3.7	266 (30.4)	1.19 (0.96, 1.48)	0.115	1.13 (0.88, 1.45)	0.348	1.13 (0.87, 1.46)	0.351	1.12 (0.87, 1.45)	0.379
Quartile 3, 3.7 to <4.2	375 (35.5)	1.50 (1.22, 1.84)	<.001	1.18 (0.93, 1.51)	0.173	1.18 (0.92, 1.51)	0.182	1.17 (0.92, 1.50)	0.207
Quartile 4, ≥4.2	403 (46.0)	2.42 (1.95, 3.00)	<.001	1.46 (1.12, 1.90)	0.0049	1.45 (1.11, 1.90)	0.006	1.42 (1.09, 1.86)	0.0101
Per 1 SD increase (0.67 mg/dL)		1.39 (1.29, 1.49)	<.001	1.10 (1.00, 1.21)	0.045	1.10 (1.00, 1.21)	0.0546	1.09 (0.99, 1.19)	0.0895
FGF23, RU/mL									
Quartile 1, <95.5	202 (21.8)	1 (Reference)		1 (Reference)		1 (Reference)		1 (Reference)	
Quartile 2, 95.5 to <145.0	281 (30.4)	1.55 (1.25, 1.93)	<.001	1.11 (0.86, 1.44)	0.424	1.09 (0.84, 1.41)	0.529	1.06 (0.82, 1.38)	0.636
Quartile 3, 145.0 to <238.0	372 (40.2)	2.42 (1.95, 3.01)	<.001	1.36 (1.05, 1.77)	0.0204	1.35 (1.03, 1.76)	0.0275	1.30 (0.99, 1.70)	0.057
Quartile 4, ≥238.0	441 (47.4)	3.29 (2.65, 4.09)	<.001	1.28 (0.97, 1.69)	0.0854	1.23 (0.92, 1.65)	0.154	1.21 (0.90, 1.63)	0.198
Per 1 SD increase^a^ (0.76)		1.52 (1.40, 1.64)	<.001	1.08 (0.99, 1.19)	0.099	1.07 (0.97, 1.18)	0.150	1.07 (0.97, 1.19)	0.161
Urine phosphate-to-creatinine ratio									
Quartile 1, <0.44	321 (35.5)	1 (Reference)		1 (Reference)		1 (Reference)		1 (Reference)	
Quartile 2, 0.44 to <0.57	317 (35.1)	1.12 (0.91, 1.37)	0.279	1.13 (0.89, 1.42)	0.318	1.13 (0.89, 1.43)	0.3167	1.12 (0.89, 1.42)	0.333
Quartile 3, 0.57 to <0.73	297 (32.9)	1.23 (0.99, 1.52)	0.057	1.28 (1.00, 1.64)	0.0458	1.28 (1.00, 1.64)	0.0477	1.27 (0.99, 1.62)	0.06
Quartile 4, ≥0.73	319 (35.2)	1.54 (1.24, 1.90)	<.001	1.52 (1.19, 1.95)	0.0009	1.51 (1.17, 1.94)	0.0013	1.48 (1.15, 1.91)	0.0022
Per 1 SD increase^a^ (0.45)		1.16 (1.08, 1.25)	<.001	1.16 (1.06, 1.27)	<.001	1.16 (1.06, 1.26)	0.0011	1.15 (1.05, 1.25)	0.0019
Phosphate burden index (Serum phosphate × parathyroid hormone)									
Quartile 1, <122.7	165 (18.2)	1 (Reference)		1 (Reference)		1 (Reference)		1 (Reference)	
Quartile 2, 122.7 to <194.8	260 (28.7)	1.71 (1.36, 2.15)	<.001	1.53 (1.18, 1.99)	0.0015	1.53 (1.17, 1.99)	0.0017	1.53 (1.17, 1.99)	0.0017
Quartile 3, 194.8 to <336.4	360 (39.7)	2.64 (2.11, 3.31)	<.001	1.96 (1.50, 2.56)	<.001	1.95 (1.49, 2.55)	<.001	1.95 (1.49, 2.55)	<.001
Quartile 4, ≥336.4	478 (52.3)	4.20 (3.34, 5.28)	<.001	2.31 (1.73, 3.08)	<.001	2.30 (1.72, 3.08)	<.001	2.30 (1.72, 3.08)	<.001
Per 1 SD increase^a^ (0.75)		1.60 (1.48, 1.73)	<.001	1.24 (1.12, 1.38)	<.001	1.24 (1.11, 1.37)	<.001	1.23 (1.10, 1.36)	<.001

*OR* odds ratio, *CI* confidence interval, *SD* standard deviation, *FGF23* fibroblast growth factor-23, *eGFR* estimated glomerular filtration rate, *CKD-EPI* chronic kidney disease epidemiology collaboration, *NSAID* nonsteroidal anti-inflammatory drug, *IL-6* interleukin-6, *TNF-α* tumor necrosis factor-alpha, *TGF-β* Transforming growth factor-beta, *BMI* body mass index

Model 1: Adjusted for age, sex, race (Black), clinic sites. Model 2: Model 1 + BMI, physical activity, weekly drinking, 24-h urinary sodium, eGFR (2021 CKD-EPI), use of diuretic, use of NSAID, aldosterone, Hemoglobin A1c. Model 3: Model 2 + log-IL-6, log-TNF-α, log-TGF-β. Model 4: Model 3 + log-FGF23 for the exposure biomarkers other than FGF23 (when FGF23 was the exposure, we adjusted for serum phosphate)

Apparent Treatment-Resistant Hypertension was defined as mean SBP ≥ 140 mm Hg or mean DBP ≥ 90 mm Hg while taking ≥3 antihypertensive medications or mean SBP < 140 mm Hg and mean DBP < 90 mm Hg while taking ≥4 antihypertensive medications at the baseline visit

^a^ Log-transformed

The highest quartile of urine phosphate-to-creatinine ratio was associated with ATRH (OR 1.48; 95% CI, 1.15-1.91), as was the highest quartile of the phosphate burden index (*serum phosphate × PTH*) (OR 2.30; 95% CI, 1.72–3.08), compared with the lowest quartile. Per one SD, analyses showed significant linear associations for the urine phosphate-to-creatinine ratio and the phosphate burden index. FGF23 demonstrated a graded association with ATRH in minimally adjusted models, but this association was attenuated and no longer significant after full adjustment.

Subgroup analysis stratified participants by eGFR ≥30 ml/min per 1.73 m^2^ versus <30 ml/min per 1.73 m^2^. Table 3 shows the OR (95% CI) for ATRH associated with levels of phosphate burden biomarkers as a continuous one SD increase. Among participants with relatively preserved kidney function (eGFR ≥30 ml/min per 1.73 m^2^), per-SD increases in serum phosphate, FGF23, urine phosphate-to-creatinine ratio, and phosphate burden index were associated with ATRH. These associations were not statistically significant among participants with eGFR <30 mL/min/1.73 m². However, the *p* values for interaction were not significant except for the phosphate burden index, suggesting that the associations did not differ across subgroups, although the phosphate burden index was more strongly associated with ATRH among participants with eGFR ≥30 mL/min/1.73 m².

**Table 3:** Multivariable-adjusted Odds Ratios for Apparent Treatment-Resistant Hypertension Per Standard Deviation Increase in Phosphate-Related Biomarkers and Phosphate Burden Index, Stratified by eGFR

	Model 1		Model 2		Model 3		Model 4	
	OR (95% CI)	*P* value for interaction	OR (95% CI)	*P* value for interaction	OR (95% CI)	*P* value for interaction	OR (95% CI)	*P* value for interaction
Serum phosphate, per 1 SD increase (0.67 mg/dL)								
eGFR <30 ml/min per 1.73 m^2^	1.21 (1.05, 1.39)	0.195	1.14 (0.96, 1.34)	0.584	1.15 (0.97, 1.36)	0.679	1.13 (0.95, 1.35)	0.690
eGFR ≥30 ml/min per 1.73 m^2^	1.38 (1.25, 1.51)		1.18 (1.06, 1.32)		1.15 (1.03, 1.29)		1.13 (1.01, 1.27)	
Log-FGF23, per 1 SD increase (0.76)								
eGFR <30 ml/min per 1.73 m^2^	1.31 (1.10, 1.57)	0.292	1.08 (0.88, 1.32)	0.497	1.10 (0.89, 1.36)	0.628	1.07 (0.86, 1.34)	0.559
eGFR ≥30 ml/min per 1.73 m^2^	1.49 (1.36, 1.63)		1.17 (1.05, 1.29)		1.13 (1.01, 1.26)		1.12 (1.00, 1.25)	
Log-urine phosphate-to-creatinine ratio, per 1 SD increase (0.45)								
eGFR <30 ml/min per 1.73 m^2^	1.01 (0.87, 1.19)	0.106	1.02 (0.86, 1.22)	0.224	1.04 (0.87, 1.24)	0.295	1.02 (0.86, 1.22)	0.298
eGFR ≥30 ml/min per 1.73 m^2^	1.21 (1.11, 1.32)		1.21 (1.09, 1.34)		1.20 (1.08, 1.32)		1.18 (1.07, 1.31)	
Log-phosphate burden index (serum phosphate × parathyroid hormone), per 1 SD increase (0.75)								
eGFR <30 ml/min per 1.73 m^2^	1.20 (1.01, 1.42)	<.001	1.13 (0.93, 1.39)	0.011	1.16 (0.94, 1.42)	0.017	1.14 (0.93, 1.41)	0.02
eGFR ≥30 ml/min per 1.73 m^2^	1.72 (1.55, 1.90)		1.45 (1.29, 1.63)		1.41 (1.25, 1.59)		1.39 (1.23, 1.57)	

*OR* odds ratio, *CI* confidence interval, *SD* standard deviation, *eGFR* estimated glomerular filtration rate, *BMI* body mass index, *CKD-EPI* chronic kidney disease epidemiology collaboration, *NSAID* nonsteroidal anti-inflammatory drug, *FGF23* fibroblast growth factor-23, *IL-6* interleukin-6, *TNF-α* tumor necrosis factor-alpha, *TGF-β* transforming growth factor-beta

Model 1: Adjusted for age, sex, race (Black), and clinic sites. Model 2: Model 1 + BMI, physical activity, weekly drinking, 24-h urinary sodium, eGFR (2021 CKD-EPI), use of diuretic, use of NSAID, aldosterone, Hemoglobin A1c. Model 3: Model 2 + log-IL-6, log-TNF-α, log-TGF-β. Model 4: Model 3 + log-FGF23 (when log-FGF23 was the exposure, we adjusted for serum phosphate)

Apparent Treatment-Resistant Hypertension was defined as mean SBP ≥ 140 mm Hg or mean DBP ≥ 90 mm Hg while taking ≥3 antihypertensive medications or mean SBP < 140 mm Hg and mean DBP < 90 mm Hg while taking ≥4 antihypertensive medications at the baseline visit

2021 CKD-EPI equation was used for estimated GFR

Sensitivity analysis using the 2025 ACC/AHA definition yielded similar associations of serum phosphate, ALP, PTH, FGF23, 24-h urine phosphate, phosphate burden index, and urine phosphate-to-creatinine ratio with ATRH (Supplementary Table 1).

## Discussion

This study demonstrated that abnormal phosphate homeostasis is associated with increased likelihood (odds) of ATRH among adult patients with CKD. A non-linear association was observed between serum phosphate and the odds of ATRH. Serum phosphate levels ≥4.2 mg/dL were significantly associated with ATRH, independent of traditional cardiovascular risk factors, kidney function, 24-h urine sodium, NSAID use, systemic inflammatory markers, RAS activation, and FGF23. Higher levels of urine phosphate-to-creatinine ratio and phosphate burden index (*serum phosphate × PTH*) showed positive dose-response associations with ATRH, suggesting that dysregulated phosphate homeostasis may contribute to this relationship, although causality cannot be determined from cross-sectional analysis. The urine phosphate-to-creatinine ratio was also positively associated with ATRH and may reflect higher dietary phosphate intake or greater systemic phosphate burden. This ratio may offer a practical alternative to 24-h urine collections in clinical assessments of phosphate burden [37].

Dysregulated phosphate homeostasis has been associated with inflammation, vascular calcification and stiffness, and endothelial dysfunction in patients with CKD [22–25]. Inflammation has been implicated in the development of hypertension [38, 39] and ATRH among patients with CKD [28]. Previous work has shown that elevated IL-6 and TNF-α and reduced TGF-β are associated with ATRH beyond traditional risk factors and kidney function [28]. In the present study, phosphate-related biomarkers were associated with ATRH independent of conventional risk factors and inflammatory markers, suggesting that additional mechanisms may contribute to phosphate-related ATRH prevalence, although causality cannot be inferred.

Vascular calcification is highly prevalent in CKD [40] and is associated with vascular stiffness and elevated phosphate levels [25, 41], which may contribute to the ATRH risk. Endothelial dysfunction, a key contributor to hypertension, is exacerbated by hyperphosphatemia [15, 16, 22, 42, 43]. Hyperphosphatemia has been associated with asymmetric dimethylarginine (ADMA) levels, potentially through phosphate-induced oxidative stress [44–49]. Collectively, prior evidence and the present findings support a potential independent role of phosphate burden in ATRH risk among individuals with CKD. Whether lowering phosphate levels toward the physiological range can improve ATRH control remains to be determined.

The non‑linear relationship observed in spline analyses suggests that phosphate may exert hemodynamic and vascular effects only after exceeding a physiological threshold. Serum phosphate is tightly regulated—including increases in PTH, FGF23, and renal phosphate excretion [9]. Once these regulatory pathways are exceeded, higher phosphate levels may promote endothelial dysfunction, vascular stiffness, and neurohormonal activation, which have been proposed in prior studies as potential contributors to ATRH [16]. This non‑linear pattern aligns with prior studies showing that higher‑normal or mildly elevated phosphate levels are associated with adverse cardiovascular outcomes [21, 50]. The observed pattern in this study should be interpreted as associative rather than causal. Further studies are warranted to confirm this finding.

This study introduced novel phosphate burden indices designed to capture the effects of phosphate on serum levels, bone metabolism (via PTH or ALP), and renal handling (via 24-hr urine phosphate, urine phosphate-to-creatinine ratio, or FEPO_4_) as serum phosphorus levels are often maintained within normal range via tight regulation and redistribution in the body. Among the indices, the phosphate burden index (*serum phosphate × PTH*), which may better reflect the effects of phosphate retention, remained significantly associated with ATRH during model selection. The association between the phosphate burden index (*serum phosphate × PTH*) and ATRH suggests potential synergistic mineral-metabolism pathways linking phosphate overload and ATRH. However, this phosphate burden index is conceptual and has not been externally validated. In humans, a large prospective study of more than 9 000 hypertensive reported that higher baseline serum phosphate levels were associated with increased SBP over five years of follow-up [50]. Animal studies have shown that high-phosphate diets increase BP through PTH-mediated renin upregulation and by augmenting the exercise pressor reflex and renal sympathetic nerve activity [12, 13]. Collectively, this index may reflect the pathological response to phosphate overload and could serve as a more sensitive marker of phosphate burden, warranting further validation in clinical studies.

FGF23 demonstrated a graded association with ATRH in minimally adjusted models, but this association was attenuated and no longer significant after full adjustment. In the Atherosclerosis Risk in Communities (ARIC) Study of 7 948 middle-aged adults without hypertension, participants in the highest decile of intact FGF23 had a higher incident hypertension compared with those in the lowest quintile, independent of kidney function, among individuals with eGFR mean (SD) 84.1 (15.5) ml/min per 1.73 m^2^ [18, 51]. Similarly, in the Coronary Artery Risk Development in Young Adults (CARDIA) Study, participants in the highest decile of C-terminal FGF23 had a 24% higher risk of developing hypertension compared with the lowest quintile among individuals with average eGFR 97.7 (16.0) ml/min per 1.73 m^2^ [19]. Both ARIC and CARDIA primarily included participants with preserved kidney function, where FGF23 may play a more prominent role in hypertension development [18, 19]. In contrast, C-terminal FGF23 was not associated with ATRH in the present CKD cohort, likely reflecting differences in study design, population characteristics, and kidney function. Further studies are needed to clarify the role of FGF23 in ATRH across varying stages of kidney dysfunction.

In subgroup analyses, serum phosphate, FGF23, the urine phosphate-to-creatinine ratio, and phosphate burden index (*serum phosphate × PTH*) were associated with higher odds of ATRH among participants with eGFR ≥30 ml/min per 1.73 m². These associations were not observed among participants with eGFR <30 ml/min per 1.73 m². This attenuation likely reflects reduced statistical power due to the smaller sample size in this subgroup. In addition, the *p* values for interaction across subgroups were not significant except for the phosphate burden index, suggesting that the associations generally did not differ across subgroups except for the phosphate burden index. The stronger association of the phosphate burden index with ATRH among participants with eGFR ≥30 mL/min/1.73 m² suggests that it may serve as an early marker of phosphate overload under pathological conditions. Future studies are needed to confirm these findings.

These findings have important clinical and public health implications, given the high prevalence of ATRH and its strong association with cardiovascular morbidity and mortality among patients with CKD [8, 9, 52]. These results support the rationale for future clinical trials to determine whether targeting phosphate burden—including maintaining serum phosphate below 4.2 mg/dL—may help prevent ATRH in patients with CKD. Future studies are needed to determine whether phosphate-related biomarkers predict clinical outcomes, improve risk stratification, or respond to interventions targeting phosphate homeostasis.

This study has several strengths. First, it includes a large CKD cohort with extensive phenotyping. Second, it provides novel evidence supporting further evaluation of phosphate burden in ATRH. Third, it introduces a new phosphate burden index that may have relevance for clinical outcomes.

However, several limitations should be acknowledged. First, the cross‑sectional design precludes temporal or causal relationships. Second, baseline data were collected between 2003 and 2008, and evolving CKD management—including the now widespread use of SGLT2 inhibitors, nonsteroidal mineralocorticoid receptor antagonists (e.g., finerenone), and angiotensin receptor–neprilysin inhibitors (sacubitril/valsartan)—may reduce the prevalence of ATRH. However, these agents are generally not considered first- or second-line therapies for resistant hypertension. In addition, finerenone and ARNI therapy are not known to directly affect phosphate metabolism. Notably, recent reports suggest that SGLT2 inhibitors may increase serum phosphate levels, which warrants further investigation regarding phosphate and blood pressure management in CKD [53]. Third, the ATRH blood pressure criteria in this study were based on older hypertension guidelines, consistent with the guidelines in place at the time of baseline data collection; however, sensitivity analyses using the 2025 ACC/AHA definition of ATRH showed similar associations. Fourth, ALP is not bone-specific; although cirrhosis was excluded, total ALP may not fully reflect bone-derived ALP. Fifth, the phosphate burden indices have not undergone external validation, and their clinical utility requires confirmation. Sixth, total PTH and C-terminal FGF23 were measured, which detect inactive fragments, which may not accurately reflect biologically active FGF23 and PTH. However, the association between the phosphate burden index, including PTH, and ATRH was independent of kidney function. Seventh, aldosterone was used as a surrogate for RAS activation, which only partially reflects RAS activity. Eighth, participants’ antihypertensive medication class, dose, and treatment intensity are not fully addressed due to a lack of data and may contribute to misclassification; however, both the number of reported medications and blood pressure levels were significantly higher in the ATRH group compared with the non-ATRH group, suggesting that phosphate-related biomarkers were associated with an uncontrolled blood pressure phenotype. Additionally, this phenotype has also been associated with an increased risk of death and cardiovascular disease events in the CRIC study.

In conclusion, higher levels of serum phosphate and phosphate‑related biomarkers—including urine phosphate-to-creatinine ratio and phosphate burden index (*serum phosphate × PTH*)—were associated with higher odds of ATRH independent of traditional risk factors, kidney function, FGF23, RAS activation, and inflammation. Further studies are warranted to determine whether targeting phosphate burden can improve ATRH among patients with CKD.

## Supplementary information

Supplementary Information
