# Supplementary material for: Phosphate homeostasis and apparent treatment-resistant hypertension in chronic kidney disease: the Chronic Renal Insufficiency Cohort (CRIC) study
Source: Hypertens Res. 2026 Jun 17;49(8):2404–16. doi: 10.1038/s41440-026-02706-5 (PMC13437241; doi:10.1038/s41440-026-02706-5)
Supplement: Supplementary file 1 [file 41440_2026_2706_MOESM1_ESM.docx]

**Supplementary Table 1. Odds Ratios (95% Confidence Intervals) for Apparent Treatment-Resistant Hypertension per 1 SD of Phosphate Biomarkers, Using the Original (≥140/90 mm Hg) and 2025 ACC/AHA (≥130/80 mm Hg) Definitions**

|  | **Outcome 1**^1^ | | | **Outcome 2**^2^ | |
| --- | --- | --- | --- | --- | --- |
|  | **OR (95% CI)** | **P value** | **OR (95% CI)** | | **P value** |
| Serum phosphate (per 1 SD increase, 0.67 mg/dL) | 1.09 (0.99, 1.19) | 0.090 | 1.08 (0.98, 1.20) | | 0.122 |
| Log-transformed alkaline phosphatase (per 1 SD increase, 0.32) | 1.03 (0.94, 1.13) | 0.492 | 1.06 (0.96, 1.17) | | 0.233 |
| Log-transformed parathyroid hormone (per 1 SD increase, 0.71) | 1.19 (1.07, 1.31) | <.001 | 1.20 (1.08, 1.34) | | <.001 |
| Log-transformed fibroblast growth factor-23 (per 1 SD increase, 0.76) | 1.07 (0.97, 1.19) | 0.161 | 1.00 (0.90, 1.11) | | 0.976 |
| 24-hour urine phosphate (per 1 SD increase, 349.1 mg) | 1.09 (0.98, 1.21) | 0.115 | 1.08 (0.97, 1.22) | | 0.172 |
| Log-transformed urine phosphate-to-creatinine ratio (per 1 SD increase, 0.45) | 1.15 (1.05, 1.25) | 0.0019 | 1.11 (1.01, 1.22) | | 0.025 |
| Log-phosphate burden index (serum phosphate × PTH) (per 1 SD increase, 0.75) | 1.23 (1.10, 1.36) | <.001 | 1.25 (1.12, 1.40) | | <.001 |
| Log-transformed fractional excretion of phosphate (per 1 SD increase, 0.52) | 1.13 (1.02, 1.24) | 0.016 | 1.09 (0.99, 1.21) | | 0.087 |

OR = odds ratio; SD = standard deviation

1 Apparent Treatment-Resistant Hypertension was defined as mean SBP ≥140 mm Hg or mean DBP ≥90 mm Hg while taking ≥3 antihypertensive medications or mean SBP <140 mm Hg and mean DBP <90 mm Hg while taking ≥4 antihypertensive medications at the baseline visit.

2 Apparent Treatment-Resistant Hypertension was defined as mean SBP ≥130 mm Hg or mean DBP ≥80 mm Hg while taking ≥3 antihypertensive medications including a diuretic or mean SBP <130 mm Hg and mean DBP <80 mm Hg while taking ≥4 antihypertensive medications at the baseline visit.

1, 2 Outcome 1 and 2 are adjusted for age, sex, race (Black), clinic sites, BMI, physical activity, weekly drinking, 24-hour urinary sodium, eGFR (2021 CKD-EPI), use of diuretic, use of NSAID, aldosterone, Hemoglobin A1c, log-IL-6, log-TNF-α, log-TGF-β, and log-FGF23 (when log-FGF23 was the exposure, we adjusted for serum phosphate).
